# Epstein-Barr virus stably confers an invasive phenotype to epithelial cells through reprogramming of the WNT pathway

**DOI:** 10.18632/oncotarget.23824

**Published:** 2018-01-02

**Authors:** Christine E. Birdwell, Kanchanjunga Prasai, Samantha Dykes, Yali Jia, Tawsha G.C. Munroe, Malgorzata Bienkowska-Haba, Rona S. Scott

**Affiliations:** ^1^ Department of Microbiology and Immunology, Louisiana State University Health Science Center-Shreveport, Shreveport, LA 71130, USA; ^2^ Feist-Weiller Cancer Center, Shreveport, LA 71130, USA; ^3^ Radiation Oncology Department, University of Florida, Gainesville, FL 32610, USA; ^4^ Current address: Department of Microbial Pathogenesis and Immunology, Texas A & M University, College Station, TX 77843, USA

**Keywords:** Epstein-Barr virus, epigenetics, WNT, LEF1, invasion

## Abstract

Epstein-Barr virus (EBV)-associated carcinomas, such as nasopharyngeal carcinoma (NPC), exhibit an undifferentiated and metastatic phenotype. To determine viral contributions involved in the invasive phenotype of EBV-associated carcinomas, EBV-infected human telomerase-immortalized normal oral keratinocytes (NOK) were investigated. EBV-infected NOK were previously shown to undergo epigenetic reprogramming involving CpG island hypermethylation and delayed responsiveness to differentiation. Here, we show that EBV-infected NOK acquired an invasive phenotype that was epigenetically retained after viral loss. The transcription factor lymphoid enhancer factor 1 (LEF1) and the secreted ligand WNT5A, expressed in NPC, were increased in EBV-infected NOK with sustained expression for more than 20 passages after viral loss. Increased LEF1 levels involved four LEF1 variants, and EBV-infected NOK showed a lack of responsiveness to β-catenin activation. Although forced expression of WNT5A and LEF1 enhanced the invasiveness of parental NOK, LEF1 knockdown reversed the invasive phenotype of EBV-infected NOK in the presence of WNT5A. Viral reprogramming of LEF1 and WNT5A was observed several passages after EBV infection, suggesting that LEF1 and WNT5A may provide a selective advantage to virally-infected cells. Our findings suggest that EBV epigenetically reprogrammed epithelial cells with features of basal, wound healing keratinocytes, with LEF1 contributing to the metastatic phenotype of EBV-associated carcinomas.

## INTRODUCTION

Nasopharyngeal carcinoma (NPC) is a squamous cell carcinoma (SCC) prevalent in Southeastern Asia and Southern China [1]. The World Health Organization has categorized NPC into three types based on the tumor's histological appearance. Type 1 is a keratinizing, differentiated SCC, type II is a non- keratinizing, differentiated SCC, and type III is a non-keratinizing, undifferentiated SCC. Epstein-Barr virus (EBV) is associated with a majority of undifferentiated NPC cases and can be found in early pre-cancerous lesions, suggesting that the virus contributes to early events in NPC progression [2–5]. Elevated EBV antibody titers are detected within three years in advance of clinical presentation and are used as a prognostic marker for NPC development [6, 7]. EBV has been associated with other undifferentiated carcinomas arising at other anatomical sites such as the stomach, tonsil, and thymus [8–10]. In EBV-associated carcinomas, a latent EBV infection is typically observed that includes expression of EBV nuclear antigen (EBNA) 1, latent membrane protein (LMP) 1, LMP2, and a number of non-coding viral transcripts (EBV encoded RNAs [EBERs] and BamHI A rightward transcripts [BARTs]) [11–13].

NPC has a poor prognosis, partly due to the advanced stage of disease where patients frequently present with lymph node metastasis at the time of diagnosis [1]. EBV has been suggested to contribute to the rapid progression and metastatic phenotype of NPC. *In vitro*, EBV-negative nasopharyngeal and gastric carcinoma cell lines infected with EBV showed increased invasiveness compared to their uninfected controls [14, 15]. Forced expression of EBV LMP1 or 2A in epithelial cells has been shown to be sufficient to enhance epithelial cell motility and invasion by various mechanisms that include upregulation of matrix metalloproteinases and acquisition of an epithelial-to-mesenchymal transition phenotype [16–20]. EBV infection of epithelial cells also affects the WNT signaling pathway, which is involved in cell differentiation, growth, survival, and movement [21–23]. In NPC, aberrant WNT signaling has been observed that includes increased expression of the WNT ligand, WNT5A, and the frizzled receptor family 7 (FZD7) [24–26]. Furthermore, LMP2A expression in epithelial cells was shown to activate WNT signaling through activation of phosphatidylinositol-3-kinase (PI3K) and the serine/threonine kinase AKT, which inactivates the negative regulator of the WNT signaling pathway, glycogen synthase kinase 3β (GSK3β), resulting in stabilization and nuclear accumulation of β-catenin [27, 28]. In nasopharyngeal cells, LMP2A expression also leads to increased WNT5A, invasion, and proliferation [26]. Furthermore, the EBV microRNAs, which are highly expressed in NPC can target the WNT signaling pathway [29, 30].

NPC has a low number of mutations; instead, NPC displays a greater DNA methylation state compared to other head and neck carcinomas [31, 32]. The tumor suppressor p53 is not commonly mutated in NPC [31]. Conversely, a number of tumor suppressors, such as p16, are silenced through DNA hypermethylation [33, 34]. DNA methylation has also been shown to silence regulators of the WNT signaling pathway, such as WNT Inhibitory Factor 1 and Adenomatous Polyposis Coli in EBV-positive carcinomas [35–37]. Such DNA hypermethylation that occurs in the promoter region of genes in GC rich stretches termed CpG islands is described as a CpG island hypermethylator phenotype (CIMP). CIMP is not unique to NPC, but can also be observed in EBV-associated gastric carcinoma [38, 39]. Such epigenetic changes in EBV-associated carcinomas may be related to viral exploitation of the host epigenetic machinery required for the establishment of viral latency. Several latent proteins are known to interact with host epigenetic modifiers to differentially regulate their activity, expression levels, or recruitment to host and viral DNA [40]. For example, LMP1 and LMP2A activation of the host DNA methyltransferases (DNMTs) results in promoter hypermethylation and silencing of the E-cadherin and Phosphatase and Tensin Homolog (PTEN) genes, respectively [41–44].

The link between EBV epigenetic modifications and phenotypic outcomes in EBV-associated carcinomas with few to no viral genes expressed at the time of clinical presentation is not fully understood. We have previously reported that transit of EBV through epithelial cells (transient infection) is associated with increased DNA methylation and CIMP in hTERT-immortalized normal oral keratinocytes (NOK). A delayed responsiveness to differentiation was also observed in EBV-positive NOK that was maintained after loss of the virus to suggests that virally-induced epigenetic changes can reprogram cellular differentiation [45]. Previously, we observed that transient EBV infection also increased the invasive phenotype of A549 lung carcinoma cells, suggesting that EBV may epigenetically reprogram infected cells with invasive potential [46]. Here, we investigated the invasive properties of NOK cells as an epigenetic event that is retained following loss of the virus. We focused on two members of the WNT signaling pathway, Lymphoid Enhancer Factor 1 (LEF1) and WNT5A, which were substantially increased after EBV infection. Increased expression of LEF1 and WNT5A is observed in NPC [26, 47].

LEF1 and WNT5A are implicated as regulators of lineage specification, differentiation, proliferation, and cell renewal, and have been linked to oncogenic phenotypes such as increased proliferation, cell motility, and invasion in epithelial cells [48, 49]. In the epithelium, LEF1 is typically expressed in progenitor cells, while WNT5A is expressed in basal epithelial cells [50, 51]. LEF1 is a member of the TCF family of transcription factors. LEF1 has no intrinsic transactivation function of its own, but instead acts upon transcription through interaction with activating co-factors such as β-catenin and Mothers Against DPP Homolog (Smad) family members or repressive co-factors like Transducin-Like Enhancer of Split (TLE) (reviewed in [52]). WNT5A is one of 19 WNT secreted ligands. WNT5A signals through a number of receptors resulting in cell-context dependent outcomes that include increased cellular motility and inactivation of canonical WNT signaling [53–55]. Here, we provide evidence that EBV-enhanced epithelial invasion was a virally-induced epigenetic event dependent on LEF1 expression. Together with previous observations that EBV infection interferes with epithelial differentiation, these data suggest that the EBV-infected oral keratinocytes acquired features of wound healing basal keratinocytes.

## RESULTS

### EBV-infected keratinocytes epigenetically maintained an invasive phenotype

We previously established EBV-positive and transiently infected EBV-negative NOK and showed epigenetic changes that resulted following EBV infection [45]. EBV-positive NOK predominantly displayed a latency I/II infection. LMP2A transcripts were detected, but not LMP1 protein. In EBV-positive NOK, Nawandar *et al*. previously reported that LMP1 expression was induced in differentiated cells [56]. We observed that passage of EBV through NOK resulted in the acquisition of global DNA methylation with evidence of CpG island DNA hypermethylation at several genes. In addition, EBV-infected NOK showed a delayed responsiveness to differentiation that was maintained after loss of the virus, a phenotype epigenetically acquired following viral infection [45].

As EBV is known to confer an invasive phenotype to infected epithelial cells [14, 15], we examined the motility and invasive potential of EBV-infected NOK, and the stability of the invasive phenotype after viral loss. Motility was measured by analysis of wound closure in real time over a 24-hour time period. Invasion was measured with two assays: 1) wound healing invasion assays measured the rate of wound closure of a population of cells through Matrigel^®^ in supplement free media conditions; and 2) transwell invasion assays measured the ability of individual cells to invade through Matrigel^®^ in response to the chemoattractant lysophosphatidic acid (LPA).

**Figure 1:**
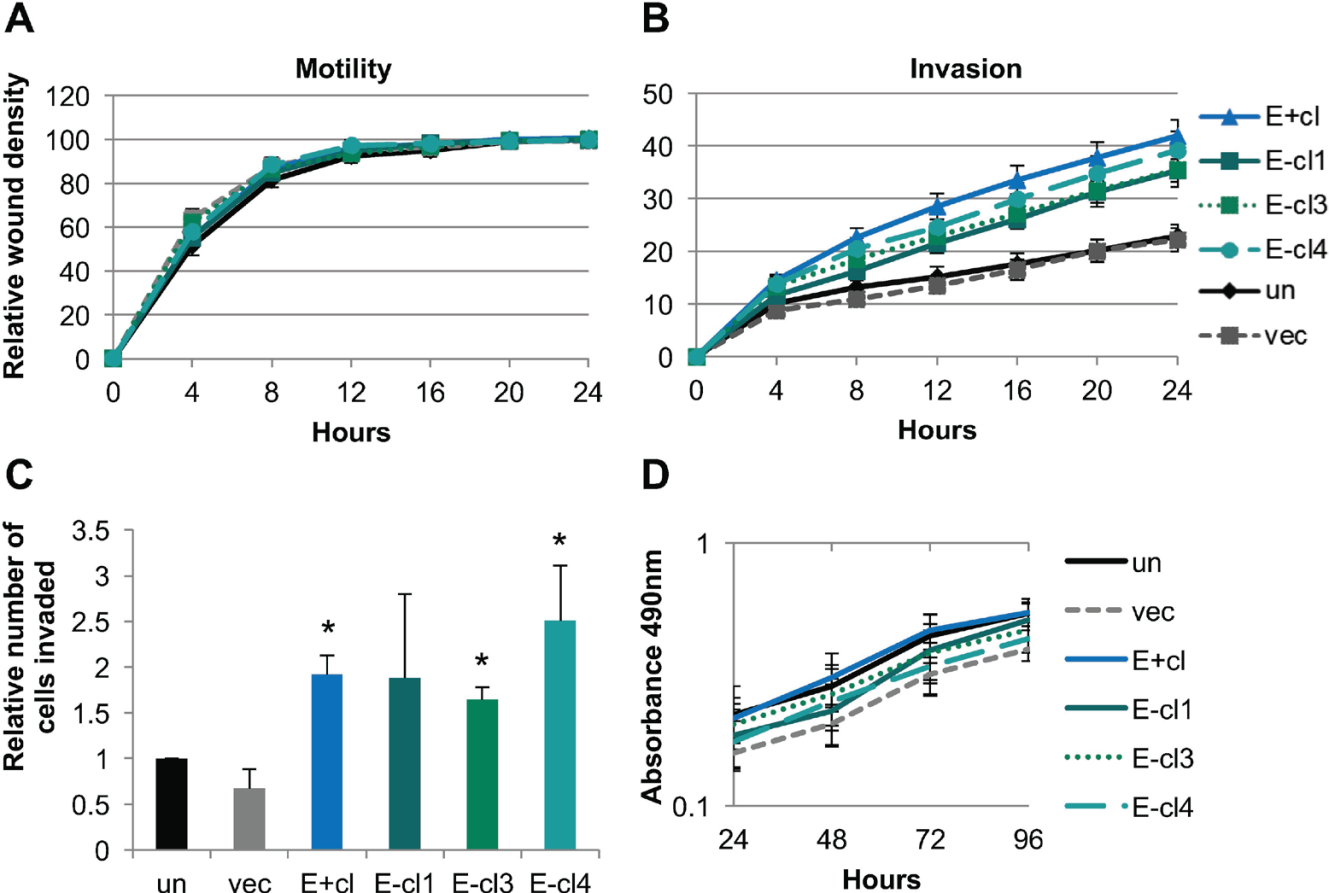
EBV-infected keratinocytes stably maintained an increased invasive phenotype (**A**) Wound healing motility assay utilizing the IncuCyte Zoom microscope to monitor rate of wound closure. Shown is the average of four biological replicates analyzed in quadruplicate. Error bars represent the standard error of the mean (SEM). (**B**) Wound healing invasion assay through 1:10 growth factor reduced Matrigel^®^ utilizing the IncuCyte Zoom microscope to measure the rate of wound closure. Shown is the average of four biological replicates each analyzed in triplicate with error bars representing the SEM. E+cl, E-cl1, E-cl3, and E-cl4 are all significantly more invasive than vector (*p* < 0.05) by area under the curve analysis followed by one-way ANOVA. (**C**) Transwell invasion assay through 1:10 Matrigel^®^ with 500 nM LPA in the bottom well as a chemoattractant. The number of migrated cells per 10× field was counted at five random locations per transwell insert. Shown is the average number of invaded cells relative to uninfected in three biological replicates each analyzed in triplicate. Error bars are the SEM with ^*^ representing *p* < 0.05 compared to uninfected. (**D)** MTS proliferation assay in supplement free media. Shown is the average and SEM absorbance values of four biological replicates each in triplicate. un: uninfected, vec: vector control, E+cl: EBV-positive clone, E-cl1/3/4: EBV-negative transiently infected clones.

Analysis of wound closure in real time showed no change in cellular motility, regardless of EBV status, as all cell lines were able to complete wound closure at similar rates (Figure 1A). In contrast, wound healing invasion assays showed that EBV-positive NOK were significantly more invasive than the uninfected controls (Figure 1B). Enhanced invasion was also observed in 3 other independently derived EBV-positive cell lines (Supplementary Figure 1C). Furthermore, EBV-negative transiently-infected cells stably maintained the increased invasive phenotype (Figure 1B, Supplementary Figure 1A). Chemotactic transwell invasion assays through Matrigel^®^ showed a greater number of invaded cells per field in the EBV-positive and EBV-negative transiently infected clones compared to uninfected and mock-infected controls (Figure 1C, Supplementary Figure 1B). Increased invasion was not due to increased cell proliferation as MTS assays showed similar growth rates over a four day span in supplement free media (Figure 1D) or under normal growth conditions as previously reported [45]. Thus, EBV infection was able to stably confer an increased invasive phenotype to keratinocytes that was not dependent on the continued presence of the virus, and suggested that invasion was related to the epigenetic cellular reprogramming that follows EBV infection.

### LEF1 and WNT5A were increased following EBV epigenetic reprogramming

**Figure 2:**
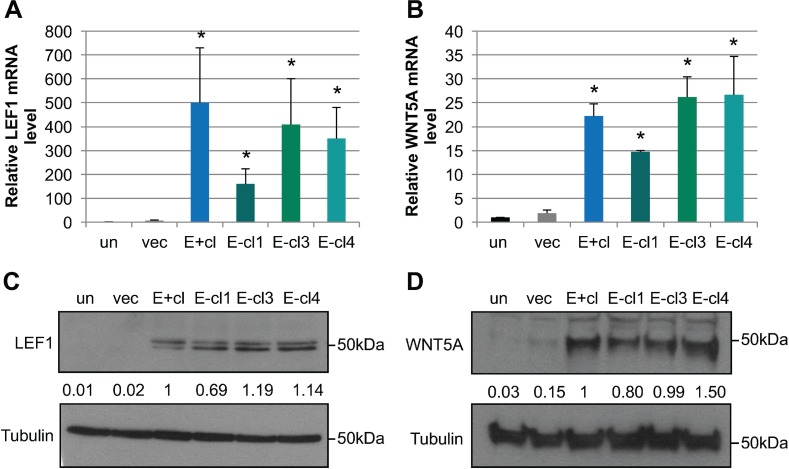
EBV-infected cells maintain increased LEF1 and WNT5A levels after loss of the viral genome mRNA levels for LEF1 **(A**) and WNT5A (**B**) were quantified by RT-qPCR relative to a standard curve. mRNA levels were normalized first to the cellular control hHPRT, and compared to the uninfected parental NOK (un). Shown is the average of three biological replicates analyzed in duplicate and SEM. Representative western blot for LEF1 (**C**) and WNT5A (**D**). Tubulin is shown as a loading control. The position of the 50 kDa molecular weight marker is shown. Signal intensity was determined on ImageJ software from at least three independent biological replicates normalized to tubulin. Averaged signal intensity values were set relative to the EBV-positive clone (E+cl). un: uninfected, vec: vector control, E+cl: EBV-positive clone, E-cl1/3/4: EBV-negative transiently infected clones (tiEBV), ^*^p value < 0.05 comparing EBV-infected to uninfected NOK controls.

To identify cellular factors that contributed to the EBV-dependent invasive phenotype, we re-analyzed our previously published microarray analysis (GSE59843) comparing the transcriptional profiles of uninfected and vector control NOK to EBV-positive and three EBV-negative transiently infected NOK clones [45]. Using Ingenuity Pathway Analysis (IPA), we identified a subset of differentially expressed genes that was associated with cellular movement (Table 1). We focused on two members of the WNT signaling pathway, LEF1 and WNT5A, with increased RNA levels in EBV-positive and EBV-negative transiently infected clones compared to uninfected controls (Figure 2A and 2B). LEF1 and WNT5A have previously been shown to enhance epithelial invasion in other cell systems [55, 57]. Compared to uninfected controls LEF1 mRNA was increased by an average of 355-fold and WNT5A mRNA was increased by an average of 23-fold in EBV-positive and EBV-negative transiently infected NOK (Figure 2A and 2B). A 100-fold increase in LEF1 protein levels and a 25-fold increase in WNT5A protein levels were observed in EBV-positive and EBV-negative transiently infected NOK clones compared to uninfected controls (Figure 2C and 2D), and suggest a transcriptional activation of LEF1 and WNT5A. Increased LEF1 and WNT5A mRNA and protein levels were observed for more than 20 passages after loss of the virus, being a stable epigenetic alteration following EBV infection of NOK (data not shown).

**Table 1:** Differentially regulated genes after EBV infection

Cellular Functions	*p*-value range	Genes
Cell-to-Cell Signaling and Interaction	1.40E-02 – 7.26E-06	ABCA1, ADAMTS20, ALS2, APLN, BST2, CAMKK2, CCL20, CCL28, CDH10, CDH11, CTSS, CXADR, CXCL1, DTNA, EMR2, EPHB2, EPO, ESM1, FER, FGF11, GGT1, HHIP, HLA-DQB1, ICAM1, IL12A, IL21R, INHA, IRS1, LEF1, NLGN1, NOS1, NPFFR2, NPR2, OCLN, OPN3, PCDHB6, PCDH10, PDPN, PTGER2, PRKAA2, RABGEF1, RPS6KA2, SAMSN1, SDC2, SERPINA1, SERPINE1, SP1, STC1, SYBU, SYK, TLR2, TLR3, TLR4, TNC, TREM2, VLDLR, WNT5A, WWC1
Cellular Movement	1.40E-02 – 5.34E-05	ABCA1, ADAMTS1, APLN, CAMKK2, CCL20, CCL28, CDH11, CTSS, CXADR, CXCL1, DNAH11, EPHB2, EPO, ESM1, F8A, FAM5C, FER, HOXA7, HTATIP2, ICAM1, IL12A, IL21R, IRS1, KAL1, LEF1, MIA, MYO5B, NOS1, OCLN, PCDH10, PDPN, PLA2R1, PLXND1, PTGER2, RABGEF1, SDC2, SERPINA1, SERPINE1, SIX4, SP1, SPOCK3, STC1, SYK, TBX3, TLR2, TLR3, TLR4, TREM2, TNC, VLDLR, WNT5A
Organismal Development	1.40E-02 – 3.14E-04	ADAMTS1, APLN, ARSB, CCL28, COL5A1, CTSS, CXADR, CXCL1, EPHB2, EPO, FAM20C, GGT1, HBB, HEXA, HLA-DQB1, HS6ST2, HTATIP2, ICAM1, IGFBP7, INHA, IRS1, LEF1, NOS1, NPR2, OCLN, PDPN, PLXND1, PRKAA2, PTGER2, PTGS1, SERPINE1, SIX4, SMAD9, SNW1, SOBP, SOHLH2, STC1, SYK, SYTL4, TBX3, TLR2, TLR3, TLR4, TNC, TREM2, VLDLR, WNT5A

Infection of NOK was performed by co-culture with EBV-positive Burkitt's lymphoma (BL) cells [45]. To ensure that the invasive phenotype and increase in LEF1 and WNT5A levels was not a result of co-culture with BL cells, NOK grown in the presence of EBV-negative BL cells were analyzed. NOK co-cultured with EBV-negative BL cells did not show any increase in invasion or LEF1 and WNT5A mRNA and protein levels over the parental uninfected population (Supplementary Figure 2), suggesting that the invasive phenotype and changes in LEF1 and WNT5A expression were due to EBV infection.

### Forced expression of LEF1 or WNT5A increased cellular invasiveness of parental NOK

**Figure 3:**
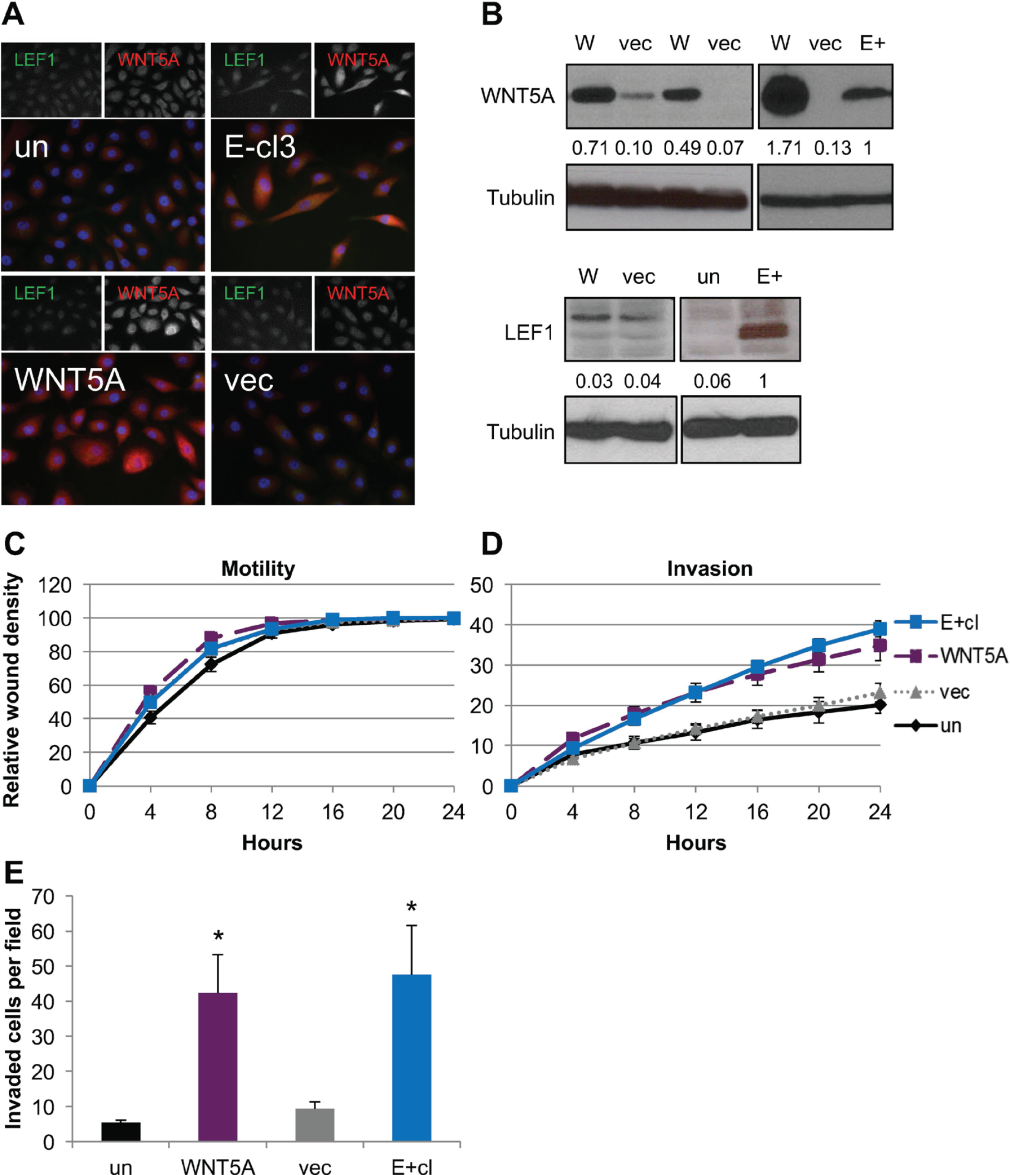
Expression of WNT5A in EBV-negative NOK conferred increased invasion (**A**) Representative immunofluorescence for WNT5A (red), LEF1 (green) and DAPI (blue). (**B**) Representative western blots for WNT5A (top) and LEF1 (bottom) with tubulin shown as a loading control. The average signal intensity of at least three biological replicates normalized to tubulin relative to the EBV-positive (E+) is shown. WNT5A stable cell lines (W), NOK transfected with empty vector (vec), uninfected, parental control NOK (un), EBV-positive NOK (E+) (**C**) Wound healing motility assay and (**D**) wound healing invasion assay for WNT5A stable cell lines compared to E+, vec, and un. Shown is the average of six biological replicates run in quadruplicate with error bars representing the SEM. WNT5A and E+ are statistically more invasive that the vector control cell lines (*p* ≤ 0.04). (**E**) Chemotactic transwell invasion assay. Shown is the average of six biological replicates analyzed in duplicate with error bars representing the SEM. ^*^p value (*p* < 0.05) relative to uninfected control (un).

Studies have shown that LEF1 or WNT5A alone can promote an invasive phenotype in epithelial cells [57, 58]. To determine if the same was true in our parental uninfected NOK cells, stable cell lines expressing either LEF1 or WNT5A were generated from uninfected parental NOK. Three independent WNT5A stable NOK cell lines and empty vector controls were generated. Two of the WNT5A stable cell lines had similar WNT5A levels as the EBV-positive cells, while a third cell line produced WNT5A at greater levels (Figure 3B). WNT5A was localized to the cytoplasm as expected in the stable cells lines (Figure 3A). Forced expression of WNT5A did not increase endogenous LEF1, as protein levels were similar to those of the vector controls (Figure 3A and 3B). In addition, no change in motility was observed between the WNT5A stable cell lines, vector control, EBV-positive or uninfected parental cells (Figure 3C). However, the stable cell lines expressing WNT5A showed an increased invasive phenotype similar to that of the EBV-positive cells in both wound healing invasion and transwell invasion assays (Figure 3D and 3E). The proliferation rate measured by MTS assays of WNT5A stable cell lines was similar to that of uninfected NOK (Supplementary Figure 3D). Thus, forced expression of WNT5A was able to confer an invasive phenotype to NOK cells.

To determine if LEF1 also contributed to the invasive phenotype in the NOK cell lines, we generated LEF1 stable cell lines and respective vector controls. LEF1 has four major transcriptional variants that are generated through alternative splicing, with each LEF1 variant potentiating different effects on cellular functions [59, 60]. LEF1 variant 1 is the full length protein (NM_016269). LEF1 variants 2, 3, and 4 have an in-frame exon VI deletion (NM_001130713, NM_001130714, and NM_001166119). LEF1 variant 3 also has a 3’ end exon insertion. LEF1 variant 4 has an N-terminal truncation with loss of the β-catenin binding site, and has been suggested to act as a dominant negative LEF1 [61]. EBV infection of NOK increased the mRNA levels of the four LEF1 mRNA variants analyzed in EBV-positive and EBV-negative transiently infected NOK by RT-PCR (Supplementary Figure 3A).

**Figure 4:**
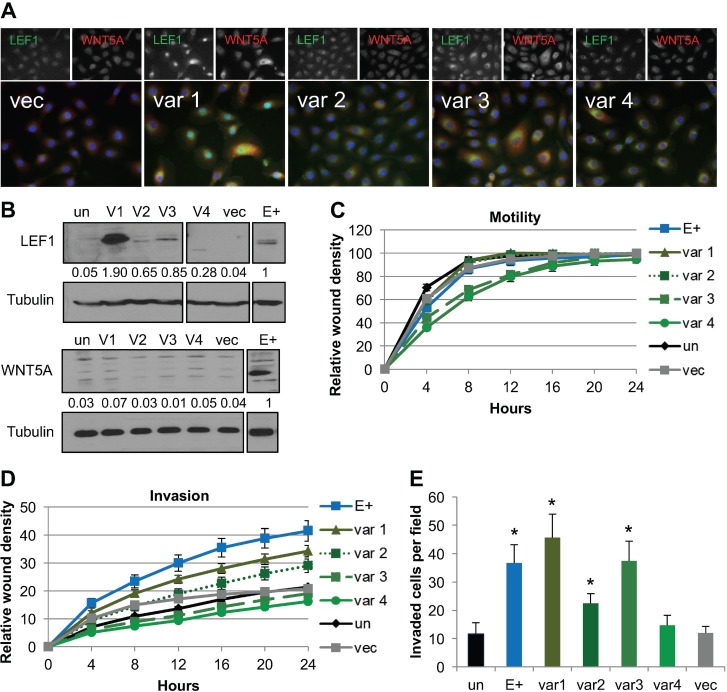
Forced expression of LEF1 variants 1, 2, and 3 enhanced invasion (**A**) Representative immunofluorescence for LEF1 and WNT5A. LEF1 is shown in green, WNT5A in red, and DAPI in blue. (**B**) Representative Western blot for LEF1 (top) and WNT5A (bottom) with tubulin shown as a loading control. Numbers are the average signal intensity of at least three biological replicates normalized to tubulin with E+ set at 1. (**C**) Wound healing motility assay. Shown is the average of three biological replicates run in quadruplicate with error bars representing the SEM. (**D**) Wound healing invasion assay. Shown is the average of three biological replicates analyzed in quadruplicate and error bars are the SEM. Using area under the curve analysis, E+ and LEF1 variant 1 were statistically more invasive than vector control cells (*p* ≤ 0.02). (**E**) Chemotactic transwell invasion assay. Shown is the average of three biological replicates analyzed in duplicate and error bars are the SEM. ^*^p ≤ 0.008 compared to un. Un: uninfected parental NOK, var1/2/3/4: LEF1 variant 1/2/3/4 transfected NOK, vec: NOK transfected with empty vector, E+: EBV-positive NOK.

To understand the contribution of each LEF1 variant to invasion, stable cell lines expressing each LEF1 variant individually were generated and characterized. Differences in LEF1 cellular localization were observed between LEF1 variants. LEF1 variant 1 protein was predominantly nuclear, with increased LEF1 protein levels compared to EBV-positive cells (Figure 4A and 4B). LEF1 variants 2, 3 and 4 all showed a predominant perinuclear localization, with protein levels of variant 2 and 3 being similar to EBV-positive cell line, while variant 4 was increased compared to the EBV-positive cell line (Figure 4A and 4B). Immunofluorescence analysis showed that forced expression of the LEF1 variants was associated with strong WNT5A signal in a subset of cells, whereas western blot analysis showed similar endogenous WNT5A levels between LEF1 stable cell lines and the uninfected and vector controls. Wound healing motility assays showed that stable cell lines expressing LEF1 variant 1 and 2 were equally motile as the control cell lines. LEF1 variants 3 and 4 were slightly less motile, yet wound closure was complete by 24 hours (Figure 4C). In wound healing invasion assays, stable cell lines expressing LEF1 variants 1 and 2 showed an increased invasive phenotype compared to the uninfected and vector controls; however, invasion was not as robust as that of the EBV-positive cells. In contrast, LEF1 variants 3 and 4 had no effect on invasion (Figure 4D). In chemotactic transwell invasion assays, LEF1 variants 1, 2, and 3 increased invasion compared to uninfected and vector control NOK, whereas LEF1 variant 4 had no effect on invasion (Figure 4D and 4E). Differences in the invasive properties of LEF1 variant 3 by wound healing versus chemotactic transwell invasion assays highlighted distinct mechanisms that govern cellular invasiveness in each assay. As LEF1 has been reported to increase proliferation of epithelial cells [58, 62], MTS assays were utilized to measure cellular growth rates to control for proliferative effects in the invasive phenotypes observed. All LEF1 stable cell lines and empty vector controls proliferated at similar rates (Supplementary Figure 3C). Together, these findings suggested that LEF1 variants 1, 2, and 3 may cooperate with WNT5A, which can act in an autocrine and paracrine manner, to increase invasion.

### LEF1 was required for the EBV-dependent invasive phenotype

**Figure 5:**
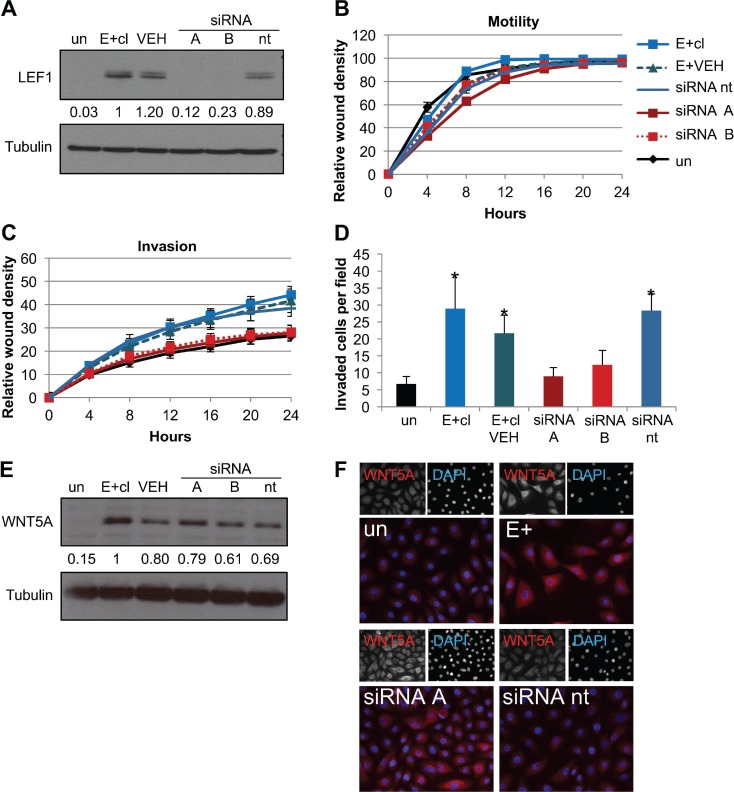
Knockdown of LEF1 reverts the EBV-dependent invasive phenotype (**A**) Representative western blot for LEF1 knockdown in E+cl cells. Tubulin is shown as a loading control. Numbers represent the average signal intensity of four biological replicates normalized to tubulin with E+cl set to 1. (**B**) Wound healing motility assay of LEF1 knockdowns in EBV-positive clone (E+cl). Shown is the average of four biological replicates analyzed in duplicate. Error bars are the SEM. (**C**) Wound healing invasion assay. Shown is the average of four biological replicates analyzed in duplicate. Error bars are the SEM. Area under the curve statistical analysis showed that uninfected and LEF1 siRNA knockdown cells were statistically less invasive than E+cl cells (*p* ≤ 0.02). (**D**) Chemotactic transwell invasion assay. Shown is the average of two biological replicates analyzed in duplicate. Invaded cells per 10× field were counted at five fields per insert. Error bars are the SEM. ^*^p ≤ 0.01 compared to un. (**E**) Representative western blot of WNT5A in E+cl LEF1 knockdown cells. Tubulin is shown as a loading control. Values are the average signal intensity of three biological replicates normalized to tubulin with E+cl set to 1. (**F**) Representative immunofluorescence of WNT5A (red) and DAPI (blue) in E+cl LEF1 knockdown cells. Un: uninfected parental cells, E+cl: EBV-positive clone, VEH: E+cl treated with transfection reagent alone, siRNA A/B: two independent siRNAs targeting LEF1, siRNA nt: non-target siRNA.

We next examined the role of LEF1 and WNT5A in promoting the invasive phenotype of EBV-positive cells. Efficient knockdown of WNT5A with either targeted short hairpin RNAs or siRNAs was not possible beyond 24 hours (Supplementary Figure 4). Such a short time frame did not allow sufficient time to complete the invasion assays. However, stable LEF1 siRNA knockdown was observed for up to a week. Four independent siRNAs specific for LEF1 were tested and the two most efficient siRNAs (A and B) were used individually producing at least 75% loss of LEF1 protein compared to EBV-positive and non-target siRNA (siRNA nt) treated cells (Figure 5A). LEF1 knockdown showed no significant effects on motility, as measured by wound healing assays, compared to EBV-positive NOK treated with a non-target control siRNA (NT), vehicle control (VEH), or untreated control (Figure 5B). In contrast, LEF1 knockdown reduced invasiveness to the level of the uninfected parental NOK in both wound healing and transwell invasion assays (Figure 5C and 5D). The reduction in invasion after LEF1 knockdown was also observed in two EBV-negative transiently infected clones (Supplementary Figure 5). The effect of LEF1 knockdown on invasion was not due to a reduction in cell viability as all cell lines showed similar levels of proliferation after 48 hours regardless of LEF1 knockdown (Supplementary Figure 3E–3G). Surprisingly, WNT5A protein levels remained elevated following LEF1 knockdown in EBV-positive cells and two EBV-negative transiently infected clones (Figure 5E and 5F, Supplementary Figure 6). These results implicate LEF1 as a key regulator of the invasive phenotype in EBV-positive and EBV-negative transiently infected cells.

### LEF1 activity in EBV-infected keratinocytes was not responsive to β-catenin

**Figure 6:**
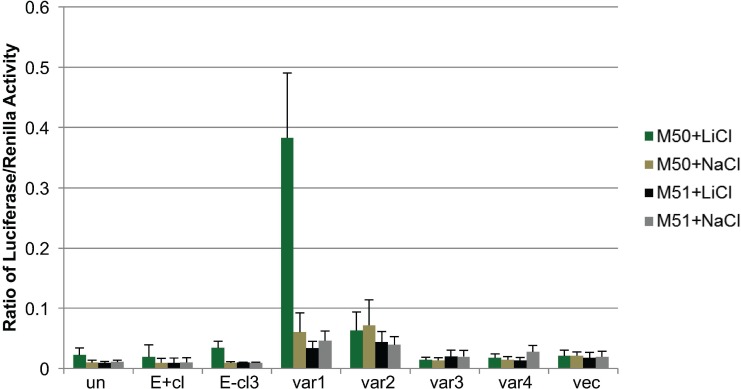
EBV reprogramming of keratinocytes results in alternative response to canonical WNT signaling Dual luciferase assay for LEF1/β-catenin activity. M50 Super 8× TOPFLASH luciferase plasmid (M50) contains 7 LEF1 response elements and the M51 Super 8× FOPFLASH plasmid (M51) contains 6 mutated LEF1 response elements upstream of luciferase reporter gene. LiCl (lithium chloride) was used to activate β-catenin and NaCl (sodium chloride) was used as a negative control. Values were normalized to renilla as a transfection control. Values are the average of four independent biological replicates and error bars are the SEM. Un: uninfected, E+cl: EBV-positive clone, var1/2/3/4: LEF1 variant 1/2/3/4 overexpressing cells, vec: vector control.

LEF1 transactivator activity is positively regulated by the co-factor β-catenin through canonical WNT signaling and has been associated with increased invasiveness in epithelial cells [55, 63]. To examine if LEF1 transactivation activity in EBV-infected NOK was dependent on β-catenin, a LEF1/β-catenin firefly luciferase reporter was used to measure LEF1 activity following activation of β-catenin. The LEF1/β-catenin firefly luciferase reporter, M50 Super 8x TOPFLASH (M50), carries 7 TCF/LEF1 binding sites upstream of the luciferase reporter. The M51 Super 8x FOPFLASH (M51) is a negative control reporter plasmid carrying 6 mutated LEF1/TCF sites [64]. Reporter plasmids were transfected into EBV-positive and uninfected parental NOK cells. A plasmid carrying a renilla luciferase expression cassette was included to normalize transfection efficiency. Lithium chloride (LiCl) was used to stabilize β-catenin by blocking GSK3β activity [65]. Accumulation of nuclear β-catenin levels was observed following LiCl treatment of NOK cells, with no change in whole cell β-catenin protein levels (Supplementary Figure 7A and 7B). Analysis of uninfected NOK cells showed no significant increase in firefly luciferase reporter activity when treated with LiCl or sodium chloride (NaCl) as a negative control, which was likely due to the low LEF1 levels in these cells. As a positive control, we used the stable cell line expressing LEF1 variant 1. When treated with LiCl, robust firefly luciferase activity was observed in cells transfected with the M50 reporter that was absent in the stable LEF1 variant 1 cells transfected with the M51 mutant reporter plasmid. In contrast, EBV-positive cells transfected with the M50 reporter plasmid showed no response to LiCl treatment, despite the relatively high levels of LEF1 protein compared to uninfected cells. The lack of response to LiCl treatment was also observed in an EBV-negative transiently infected clone as well as in stable cell lines expressing LEF1 variants 2, 3, or 4 (Figure 6). Renilla luciferase levels were similar in all cell lines (Supplementary Figure 7C). These results demonstrated that the LEF1 activity in EBV-infected NOK was not responsive to β-catenin activation, further suggesting that LEF1 enhancement of invasion is independent of β-catenin.

### LEF1 activity in EBV-infected keratinocytes was independent of AKT or NF-κB activity

**Figure 7:**
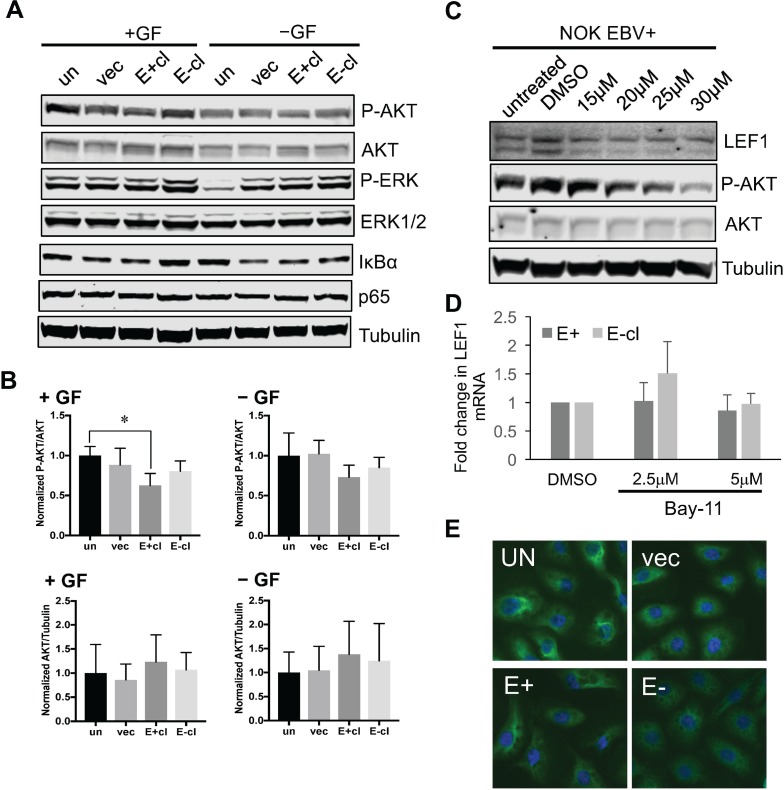
Upregulation of LEF1 in EBV-infected NOK is independent of AKT or NF-kB activity (**A**) Analysis of AKT, ERK, and NF-kB activation in EBV-positive NOK in the presence (+GF) or absence (-GF) of growth factors supplements. Representative western blots are shown examining phosphorylation of AKT (P-AKT S473); and ERK (P-ERK T202/Y204). The levels of the IkBα inhibitor of NF-kB is also shown. The total protein levels of AKT, ERK, and p65 are shown. Tubulin served as an additional loading control. (**B**) The ratio of P-AKT to total AKT (top panels) and total AKT to tubulin (bottom panels) is shown. (**C**) Effect of the PI3K inhibitor, LY294002, on LEF1 protein levels. Increasing concentration of LY294002 was used and the levels of LEF1 protein were measured. The level of P-AKT was used as an indicator for inhibition of AKT activity. Shown are representative blots for LEF1, P-AKT, AKT, and tubulin. (**D**) Effect of Bay11 on LEF1 transcript levels. qRT-PCR was used to measure LEF1 transcript levels with 2.5 and 5 uM of Bay11. (**E**) Immunofluorescence to visualize p65 localization. Un: uninfected, parental NOK, vec: vector control, E+cl: EBV-positive NOK, E-cl: EBV-negative transiently infected cl3.

Previous studies have shown that EBV LMP1 and LMP2A enhanced cell migration and invasion through activation of various pathways that included ERK-MAPK or AKT pathways [19, 66–68]. Furthermore, LMP2A has been implicated in activation of the WNT signaling pathway through the activation of AKT [27, 28]. Thus, we investigated if EBV-infected NOK showed alterations in AKT or MAPK signaling pathway that together with LEF1 could potentiate cell invasion. Cells were grown in media supplemented with growth factors (+GF) or growth factor starved for 24 hours (−GF). In either condition, we did not observe an increase in phosphorylated AKT (Ser473) or ERK (T202/Y204) in EBV-positive and transiently infected EBV-negative NOK compared to uninfected controls (Figure 7A). A decrease in phosphorylated AKT was observed in EBV-positive NOK that may reflect the slight increase in the steady state levels of AKT (Figure 7B).

Hepatocyte growth factor (HGF) transcriptional activation of LEF1 was shown to be dependent on AKT/NF-κB signaling in liver and breast carcinoma cell lines [69]. The NF-κB p65 subunit was also shown to bind the LEF1 promoter [69]. To examine if either AKT or NF-κB was involved in the upregulation of LEF1 expression in infected NOK, cells were treated with LY294002, an inhibitor of the PI3K/AKT signaling, or BAY11-7082, an inhibitor of NF-κB activation. Addition of either LY294002 or BAY11 did not reduce LEF1 RNA or protein levels in EBV-positive NOK (Figure 7C and 7D). In addition, NF-κB activation was not apparent as IkBα levels and nuclear levels of the p65 NF-κB subunit were similar between infected and uninfected controls (Figure 7A and 7E). Thus, the LEF1 RNA and protein levels observed following EBV infection were independent of AKT and p65 NF-κB activity.

### LEF1 and WNT5A RNA levels increased late after EBV infection of keratinocytes

**Figure 8:**
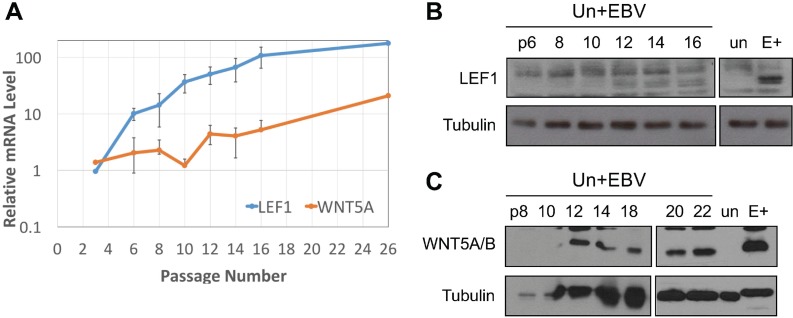
LEF1 and WNT5A upregulation are late events after EBV infection of keratinocytes (**A**) RT-qPCR for LEF1 (blue) and WNT5A (orange) in EBV-infected NOK over serial passages. Values are the average fold increase over uninfected of three biological replicates (except for the final point which is one replicate) run in duplicate. Error bars are the SEM. Representative western blot for (**B**) LEF1 and (**C**) WNT5A in EBV-infected NOK over indicated serial passages (numbers). Tubulin is shown as a loading control. Un+EBV: NOK newly infected with EBV, E+: EBV-positive NOK late passage, un: uninfected parental NOK.

A previous study showed that forced expression of LMP2A dramatically increased WNT5A levels in nasopharyngeal cell lines [26]. In the NOK cell line, transient or stable transfectants expressing LMP2A failed to increase LEF1 or WNT5A mRNA levels (data not shown). In addition, increased LEF1 or WNT5A expression was not detected within the first week of infection. However, 4 independently derived EBV-infected cell lines reproducibly showed increased LEF1 after selection and establishment of cell lines (Supplementary Figure 3B). To determine the timing of WNT5A and LEF1 reprogramming following EBV infection, NOK cell lines were infected with a recombinant EBV bearing a neomycin resistance cassette and GFP marker. EBV-positive cells were sorted by GFP expression and maintained on selection to ensure the continued maintenance of the viral episome. The experiment was repeated three times with RNA and protein harvested at various passages following infection. LEF1 and WNT5A mRNA levels were similar to uninfected parental controls through the first 3 passages. LEF1 mRNA levels began to increase 6 passages after EBV infection and could be weakly detected by western blot around passage 8 (Figure 8A and 8B). The level of LEF1 mRNA and protein continued to rise with each subsequent passage. The increase in WNT5A mRNA appeared around passage 10, with the timing being delayed in relation to the increase in LEF1 mRNA. As EBV-infected cell lines were sorted on the basis of GFP, the absence of signal through the first 3 passages argues against clonal selection of a population of cells already expressing LEF1 and WNT5A. Instead, these results suggest that EBV infection induced a slow cellular reprogramming that resulted in selection of stable expression of LEF1 and WNT5A that remained even after loss of the virus. Overall, the data presented here suggests that EBV infection of epithelial cells contributes to tumor progression and metastasis through selection of virally-induced epigenetic phenotypes that would be maintained independently of viral gene expression or the continued presence of viral genome.

## DISCUSSION

In this study, hTERT-immortalized normal oral keratinocytes (NOK) infected with EBV were investigated to decipher functional consequences that follow EBV infection. We observed that EBV infection reprogrammed keratinocytes with an invasive, wound healing phenotype that was dependent on the WNT signaling pathway. EBV viral gene expression was not required as the invasive phenotype was maintained following loss of the viral genome (Figure 1). The transiently infected EBV-negative clones (E-cl1/3/4) used in this study had lost the virus for 20 or more passages, but still maintained the invasive phenotype suggesting an epigenetic inheritance of invasion after EBV infection. There was no change in the motility of NOK cell lines regardless of viral status. Previously, we showed that EBV cellular reprogramming following infection of NOK also delayed differentiation in response to calcium or upon suspension in methylcellulose [45]. Together, these data suggested that EBV-infected NOK phenotypically acquired features of basal, wound healing keratinocytes reminiscent of the undifferentiated and metastatic phenotype noted for EBV-associated NPC.

The invasive phenotype was present in the absence of viral gene expression and viral DNA in EBV-negative transiently infected clones, suggesting a dependence on cellular gene expression, specifically LEF1, for maintenance of the epigenetically reprogrammed state. The WNT signaling pathway is involved in the regulation of cell movement and differentiation [70, 71], with WNT5A and LEF1 both shown to enhance cellular migration, proliferation, and invasion [53, 57, 58, 72]. We demonstrated that forced expression of WNT5A or LEF1 in the uninfected parental NOK cell line increased cell invasion, with WNT5A able to enhance invasion to similar levels seen following EBV infection (Figure 3 and 4). Surprisingly, in EBV-positive and EBV-negative transiently infected NOK, LEF1 was a key regulator of the EBV-induced invasive phenotype despite high levels of WNT5A (Figure 5E and 5F). In addition, we observed that upregulation of LEF1 following EBV infection or after forced expression of LEF1 variants 1, 3, and 4 increased WNT5A in select cells. These findings differ from what is observed in melanoma, where upregulation LEF1 was associated with a non-invasive, proliferative phenotype, while knockdown of LEF1 in melanoma increased WNT5A [73]. Such context-dependent differences have been previously described for WNT5A and LEF1 in their modulation of invasion (reviewed in [71, 74, 75], and may be influenced by the relative expression of LEF1 variants and other gene expression changes in the cell.

LEF1 is well known to mediate the nuclear response to WNT activation and signaling. In the absence of activating signals, LEF1 associates with Groucho/TLE repressors and inhibits gene expression [52]. Nuclear β-catenin binds to LEF1, displacing the Groucho/TLE repressors, resulting in transcriptional activation of downstream target genes (c-MYC, Cyclin D1, and LEF1) in response to activation of canonical WNT signaling [76]. *In vivo*, EBV-positive gastric carcinomas show increased β-catenin nuclear localization versus EBV-negative gastric carcinoma and β-catenin levels are increased in a majority of NPC [77, 78]. Signaling by the EBV oncoprotein LMP2A in epithelial cells has been shown to inhibit the negative regulator of β-catenin, GSK3β, leading to β-catenin stabilization and nuclear accumulation, but can also induce the non-canonical WNT ligand, WNT5A, in nasopharyngeal epithelial cells. Thus, EBV is capable of modulating both canonical and non-canonical WNT signaling [26, 27]. In the uninfected parental cell lines, stable expression full length LEF1 (variant 1) led to β-catenin activation of an LEF1 responsive luciferase reporter. In contrast, EBV-positive NOK and LEF1 variants 2, 3, and 4 were insensitive to LiCl-activation of WNT/β-catenin signaling in a LEF1 luciferase reporter assay (Figure 6). In addition, LEF1 target genes such as c-MYC or Cyclin D1 were not upregulated in the EBV-positive or transiently infected EBV-negative clones compared to uninfected controls (data not shown). Together, these results suggest that the increased LEF1 following EBV infection was not responsive to the canonical WNT signaling pathway, which may be due to a block β-catenin activation by the LEF1 variants or to WNT5A activation of the non-canonical WNT pathway [52]. We also observed no changes in the overall activation of AKT and ERK in EBV-positive and transiently-infected EBV-negative NOK compared to uninfected NOK. However, all NOK cell lines were motile, filling in a wound by 24 hours. This motile phenotype is likely due to activation of AKT and ERK signaling pathways, evidenced by the presence of their phosphorylated forms (Figure 7).

LEF1 variants have been shown to have different effects on target gene expression, proliferation and cell migration of pancreatic tumor cell lines [59]. Thus, the inability of EBV-infected cells to respond to β-catenin activation could also be explained by the concurrent expression of multiple LEF1 variants. Forced expression of LEF1 variants 2, 3, and 4 showed perinuclear localization by immunofluorescence (Figure 4A), while full length LEF1 variant 1 resided primarily in the nucleus. It is not clear what functions LEF1 variants play outside of the nucleus, but cytoplasmic localization of LEF1 has been observed following expression of Hepatitis B virus surface antigen in hepatocytes [79]. Although LEF1 variants 2 and 3 were predominantly perinuclear, cells stably expressing these variants still showed increased invasiveness, suggesting that LEF1 variants may regulate invasion by sequestering repressive factors in the cytoplasm. Alternatively, the increased abundance of LEF1 variants may result in their nuclear accumulation and activation of the invasive phenotype. In addition, each LEF1 variant may associate with different transcriptional repressors or co-activators to regulate the reprogrammed gene expression state observed following EBV infection.

Upregulation of LEF1 and WNT5A expression was observed 3 passages after EBV infection (Figure 8). Routine passaging and selection of NOK does not result in an upregulation of LEF1 and WNT5A as observed following EBV infection. The delayed upregulation of LEF1 and WNT5A following EBV infection suggests positive selection of cells reprogrammed with increased WNT5A and LEF1 levels. The EBV lifecycle is tuned to the epithelial differentiation state, replicating in the differentiated upper stratified layers of the epithelium [80]. LEF1 and WNT5A are naturally expressed in less differentiated epithelial progenitor cells or in basal epithelial cells [50, 51]. In addition, EBV-associated carcinomas, such as NPC, which are undifferentiated tumors that carry a latent viral infection, have been associated with increased LEF1 and WNT5A mRNA levels [26]. Together these observations suggest that increased levels of LEF1 and WNT5A following EBV infection reprograms epithelial cells to a less differentiated state that may promote viral latency rather than replication. Future studies will be directed at the possible role of LEF1 in maintaining EBV latency.

We have shown that EBV-infected NOK acquired features of basal, wound healing keratinocytes. In addition, our data implicate epigenetic reprogramming of the WNT pathway as an EBV-dependent driver of the invasive phenotype in epithelial cells. As such, the EBV-dependent invasive phenotype required the WNT transcription factor LEF1, potentially acting in a non-canonical manner. We have identified LEF1 as a potential biomarker and therapeutic target for the metastatic phenotype of EBV-associated carcinomas. Understanding EBV-induced epigenetic reprogramming provides a model system to decipher the complex interactions of the WNT pathway that are frequently altered in cancer. Such EBV-induced epigenetic changes also provides a framework for EBV “hit-and-run” oncogenesis in epithelial cells with long term effects on tumor progression in the context of viral latency where few or no viral genes are expressed.

## MATERIALS AND METHODS

### Cell culture

A clonal cell line derived from human telomerase reverse transcriptase (hTERT)-immortalized normal oral keratinocytes (NOK, kindly gifted by Dr. Karl Münger [81]) was infected with EBV through co-culture with the EBV-positive Akata BL cell line. EBV-negative transiently infected clones were established as previously described [45]. Uninfected controls included the parental NOK cell line, NOK stably transfected with a vector carrying neomycin resistance cassette (vec) and in some cases NOK co-cultured with EBV-negative Akata BL cells (mock infected) (Supplementary Figure 2). We previously excluded the presence of EBV genetic elements by sampling the EBV genome by PCR and southern blotting in EBV-negative transiently infected clones, verified cell lineage by short tandem repeat analysis and excluded the presence of mycoplasma in our cultures [45]. We have also excluded contamination with squirrel monkey retroviruses previously described to be a transmissible viral contaminant found in some EBV-infected cell lines [82–84]. In addition, the status of p53 gene in the NOK lines was verified by sequencing as wildtype with a heterozygous polymorphism at P72R. The clonal NOK cell line was used for forced expression of LEF1 and WNT5A. EBV-positive and stable NOK cell lines were selected and maintained with 350 μg/mL G418.

### Reverse transcription quantitative PCR (RT-qPCR)

Total cellular RNA was isolated, and cDNA was generated using SuperScript III or IV as recommended by the manufacturer. RT-qPCR was performed on a 7500 FAST Applied Biosystems thermocycler using Power SYBR Green (Life Technologies), 50 ng of cDNA, and 300 nM primers in each reaction. Relative RNA levels were determined using standard curve analysis based on serially diluted cDNA derived from clonal EBV-positive (E+cl) NOK cells or the clonal uninfected cell line as required. The cellular housekeeping gene hypoxanthine-guanine phosphoribosyl transferase (hHPRT) or cyclophillin A (Cyc) was used to as a normalization control. Negative controls included reverse transcriptase-negative reactions and water as template. Primers are listed in Supplementary Table 1.

### Chemotactic transwell invasion assays

Chemotactic transwell invasion assays were performed in duplicate. The interior of the inserts (Corning) were coated with 50 μL of 1:10 growth factor reduced Matrigel^®^ (Corning) and allowed to set at 37°C for at least 1 hour. 3 × 10^4^ cells/insert were seeded on top of the Matrigel^®^ in supplement free KSFM. The bottom well contained complete media plus 500 nM lysophosphatidic acid (LPA). Cell invasion assays were incubated for 24 hours at 37°C, washed with cold Phosphate Buffered Saline (PBS), and fixed in cold 4% paraformaldehyde (PFA) for 20 minutes. Inserts were stained with 0.2% crystal violet for 25 minutes, washed three times with distilled deionized water and cells that did not invade were removed from the interior of the insert with a sterile cotton swab. Five pictures of each insert were imaged on the Olympus DP71 microscope at 10× magnification and invaded cells were counted utilizing the ImageJ software.

### Wound healing motility and invasion assays

4 × 10^4^ cells/well were seeded in duplicate or quadruplicate in a 96 well Imagelock plate (EssenBioscience). 16–18 hours post seeding, cell monolayers were wounded using the Cell Wounder 96 (EssenBioscience) and washed twice with PBS. For invasion, 50 μL of a 1:10 growth factor reduced Matrigel^®^ (Corning) in supplement free KSFM solution was overlaid followed by an additional 100 μL of supplement free KSFM media once the Matrigel^®^ had set (10 to 20 minutes at 37°C). For motility assays, following monolayer wounding, 100 μL of supplemented KSFM was added. Plates were placed in the IncuCyte Zoom (EssenBioscience) incubator microscope and pictures taken with 10× objective every four hours. Data was analyzed with the IncuCyte Zoom software (EssenBioscience), which calculated the relative wound density defined as the density of cells within the wound as a percent of cell density of the monolayer at the starting time. Statistical analysis was performed by determining the area under the curve for each replicate followed by one-way ANOVA compared against the vector control cell line.

### Western blot

Protein lysates were collected in reducing 1× NuPage Buffer or non-reducing 1× NuPage Buffer (minus dithiothreitol [DTT]). For LEF1 and WNT5A western blots, samples were boiled for three and a half minutes before equal volumes were loaded in 10% SDS-PAGE gels and run at constant amperage. Gels were then transferred for two hours at 50 volts onto 0.45 μM PDVF membranes (Millipore) in 20% methanol transfer buffer. Membranes were blocked for one hour at room temperature in 5% milk and 5% BSA Tris buffered saline Tween-20 (TBST). Primary antibodies were hybridized overnight at 4°C in blocking buffer. After 3 TBST washes, secondary antibody in blocking buffer was added for one hour at room temperature. Following 3 TBST washes, blots were visualized using chemiluminescence (Pierce ECL2, Thermo).

Fluorescent western blotting was also used for detection. Protein extracts were collected in 1× NuPage Buffer containing DTT. Following incubation at 70°C for 10 minutes, equal volumes of samples were loaded in 10% SDS-PAGE gels and electrophoresed at constant voltage. Protein extracts were then transferred to PVDF membranes at 90 volts for 75 minutes. Membranes were blocked with Odyssey Blocking Buffer (LI-COR) at room temperature for 1 hour before incubating with the indicated primary antibodies overnight at 4°C. Following 4 TBST washes, Odyssey secondary antibodies (goat anti-rabbit IRDye 800CW and/or goat anti-mouse IRDye 680RD; dilution 1:15000) were applied for 1 hour at room temperature. After 4 TBST washes, blots were imaged using an Odyssey Infrared Imaging System (LI-COR). Scan resolution of the instrument ranges from 21 to 337 μm, and in this study blots were imaged at 169 μm. Quantitation of fluorescent signals was performed on single channels using Image Studio Lite software (LI-COR) according to the manufacturer's instructions. Antibodies used are listed in Supplementary Table 2.

### Cell proliferation assays

Cell proliferation was determined using the Promega CellTiter 96^®^ AQueous One Solution as previously described [45]. Absorbance was read on a FLUstar Omega (BMG Labtech) plate reader at 490 nm. For LEF1 knockdowns, cells were seeded three days post transfection in supplement free KSFM and metabolic activity was measured at 48 hours.

### siRNA knockdown

Cells were seeded at 2 × 10^5^ cells/well in 12 well plates. The following day cells were transfected with 10mM siRNA (Dharmacon) and 5 μL/mL Dharmafect 1 (Dharmacon) in a total of 600 μL KSFM for five hours following 15 minutes incubation on ice. Three days post siRNA transfection, cells were harvested and re-seeded for invasion and motility assays as described above. Two independent LEF1 siRNAs (Dharmacon, NCBI Probe ID 12218478 and 12218481) and a non-target siRNA (Dharmacon, D-001810-01-05) were used. Vehicle (VEH) control was treated with the transfection reagent Dharmafect without any siRNA.

### Transfection for generation of stable cell lines

1 μg of plasmid DNA (LEF1: Origene, WNT5A: Addgene Supplementary Table 2), 5 μL/mL Dharmafect 1, in 600 μL of KSFM media was allowed to incubate on ice for 15 minutes before being added to cells for 5 hours. On the third day, 350 μg/mL of G418 was added. Stable cell lines generated consisted of pools of 2 more clones and were maintained on continuous selection.

### Immunofluorescence

1 × 10^5^ cells/well were seeded onto microscope coverslips. 48 hours post seeding, cells were fixed for 20 minutes with ice cold 4% PFA, permeabilized with 0.2% Triton X-100 for 10 minutes, and blocked in 5% goat serum in PBS for 30 minutes. Primary antibody (1:100 for LEF1 and WNT5A Supplementary Table 2) was incubated overnight in BSA Saponin (BSP) blocking buffer at 4°C. Slides were washed in PBS and secondary antibody was added (1:750 anti-rat [Thermo Fisher] and 1:100 anti-rabbit [Jackson IR]) for 1 hour at room temperature. For p65 staining, PFA fixed cells were permeabilized with 5% BSA + 0.5% Triton X-100, quenched endogenous peroxidases for 30 minutes at RT (3% hydrogen peroxide), and blocked for 1 hr at RT (1% BSA + 0.5% Triton X-100). Slides were incubated with primary antibody (1:50 dilution, NF-kB p65 (F-6), Santa Cruz) overnight, washed with PBS, followed by incubation with a poly-HRP conjugated antibody for 1 hour and washed with PBS. P65 was detected with tyramide per manufacturer's instructions. After further PBS washes, slides were DAPI stained and mounted. Images were taken with a 20× objective on an Olympus Bx50 fluorescent microscope equipped with a SenSys camera system. Images were analyzed using the ImageJ software.

### Luciferase reporter assays

1.25 × 10^5^ cells/well were seeded in a 24 well plate. The following day, firefly luciferase constructs M50 Super 8× TOPFLASH (Addgene 12456) with 7 LEF1/β-catenin binding sites or the M51 Super 8× FOPFLASH (Addgene 12457) with 6 mutated LEF1/β-catenin binding sites were transfected at 7:1 ratio to a control renilla luciferase plasmid. For transfection, 1 μL polyethylenimine (PEI, 2 mg/mL) and 500 ng of total DNA were mixed with 600 μL of KSFM and incubated at room temperature for 10 minutes. Cells were transfected overnight. Six hours before harvest, 20 mM of lithium chloride (LiCl) or control sodium chloride (NaCl) was added. Lysates were collected with the Dual-Luciferase Reporter System (Promega) per the manufacturer's instructions. Firefly luciferase and renilla luciferase activity were assayed on a FLUstar Omega (BMG Labtech) plate reader sequentially for 10 seconds each. Firefly luciferase values were normalized to renilla luciferase as a transfection efficiency control.

## SUPPLEMENTARY MATERIALS FIGURES AND TABLES



## Abbreviations

ANOVA: Analysis of variance
cDNA: complementary DNA
CIMP: CpG island hypermethylator phenotype
Cyc: Cyclophillin A
EBV: Epstein-Barr virus
hHPRT: human hypoxanthine-guanine phosphoribosyl transferase
hTERT: human telomerase reverse transcriptase
LEF1: lymphoid enhancer factor 1
LMP: latent membrane protein
NOK: h TERT-immortalized normal oral keratinocyte
NPC: nasopharyngeal carcinoma
tiEBV: EBV-negative transiently infected
WNT: Wingless-Type MMTV Integration Site Family
WNT5A: Wingless-Type MMTV Integration Site Family, Member 5A
