# Supplementary material for: Epstein-Barr virus stably confers an invasive phenotype to epithelial cells through reprogramming of the WNT pathway
Source: Oncotarget. 2018 Jan 2;9(12):10417–35. doi: 10.18632/oncotarget.23824 (PMC5828208; doi:10.18632/oncotarget.23824)
Supplement: Supplementary file 1 [file oncotarget-09-10417-s001.pdf]

Epstein-Barr virus stably confers an invasive phenotype to epithelial cells through reprogramming of the WNT pathway

SUPPLEMENTARY MATERIALS

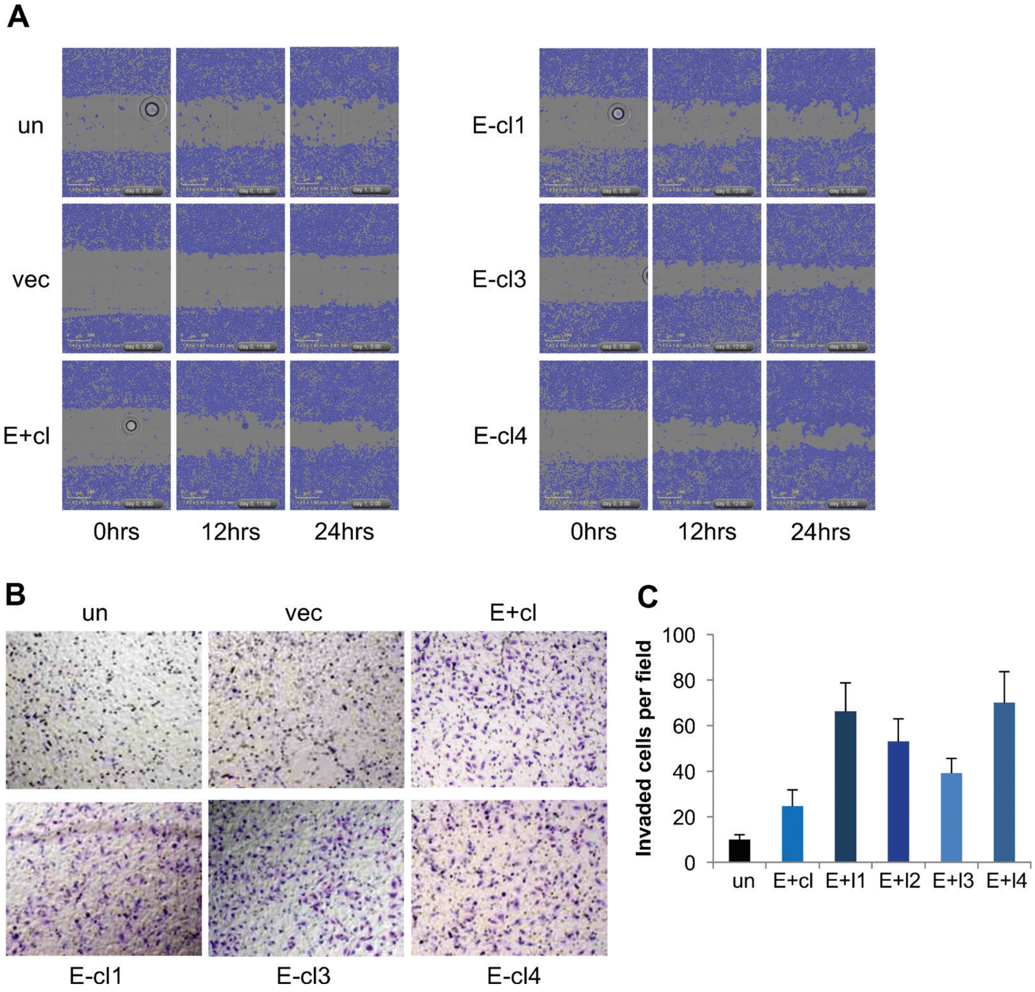

Supplementary Figure 1: Increased invasiveness is stable after EBV infection of NOK. (**A**) Representative images of wound healing invasion assay pseudo-colored with IncuCyte Zoom software. (**B**) Representative images of transwell invasion assay. Contrast was enhanced uniformly in ImageJ. (**C**) Transwell invasion assay for four independent EBV-positive cell lines at 24 hours. Shown is the average of two biological replicates analyzed in duplicate. Error bars are the SEM. Un: uninfected cells, vec: vector control cells, E+cl: EBV-positive clone, E-cl1/3/4: EBV-negative transiently infected clones, E+I1/2/3/4: EBV-positive cell lines.

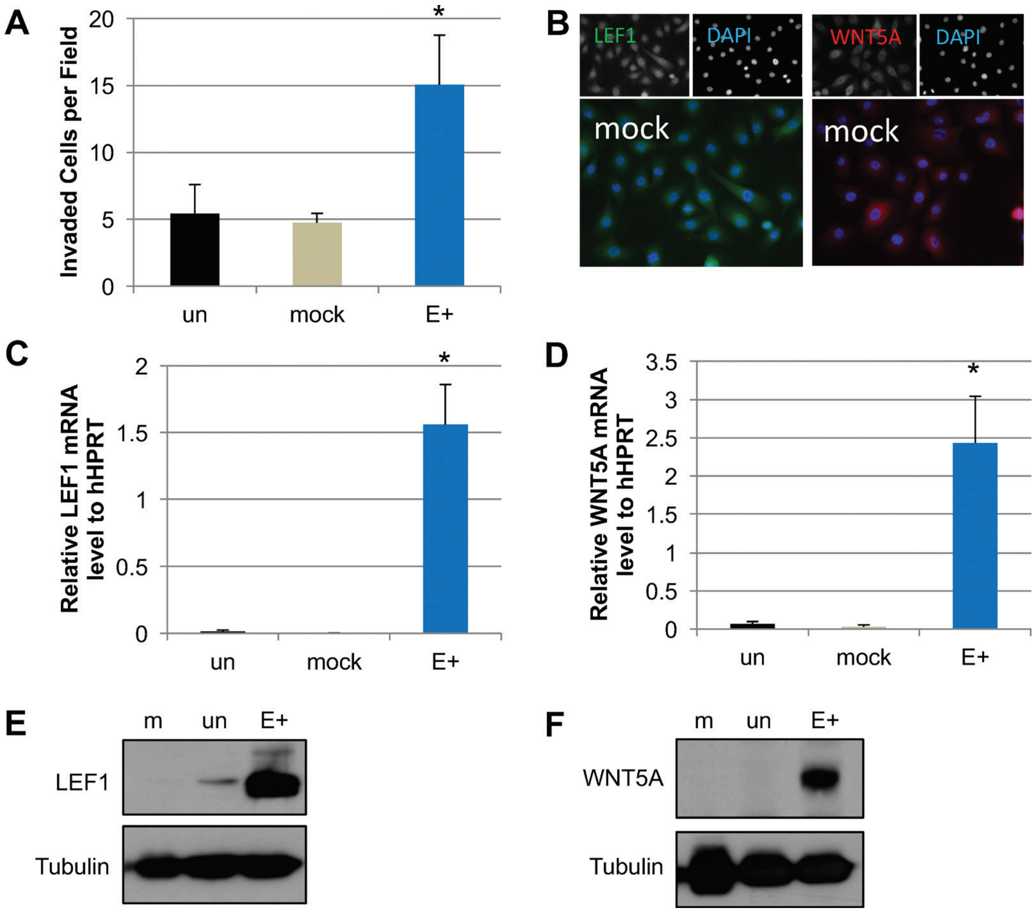

Supplementary Figure 2: Co-culture with EBV-negative Burkitt's lymphoma cells does not confer invasiveness and upregulation of WNT pathway in NOK. (**A**) Transwell invasion assay. Values are the average of four biological replicates analyzed in duplicate and error bars represent the SEM. (**B**) Representative immunofluorescence for LEF1 (green) and WNT5A (red) in mock EBV infected cells. DAPI is shown in blue. Relative mRNA levels of (**C**) LEF1 and (**D**) WNT5A to cellular control hHPRT by RT-qPCR. Values are the average of four biological replicates analyzed in duplicate and error bars are the SEM. Representative western blots for (**E**) LEF1 and (**F**) WNT5A with tubulin shown as a loading control. Un: uninfected cells, mock: mock EBV-infected cells co-cultured with EBV-negative B cells, E+: EBV-positive cells.

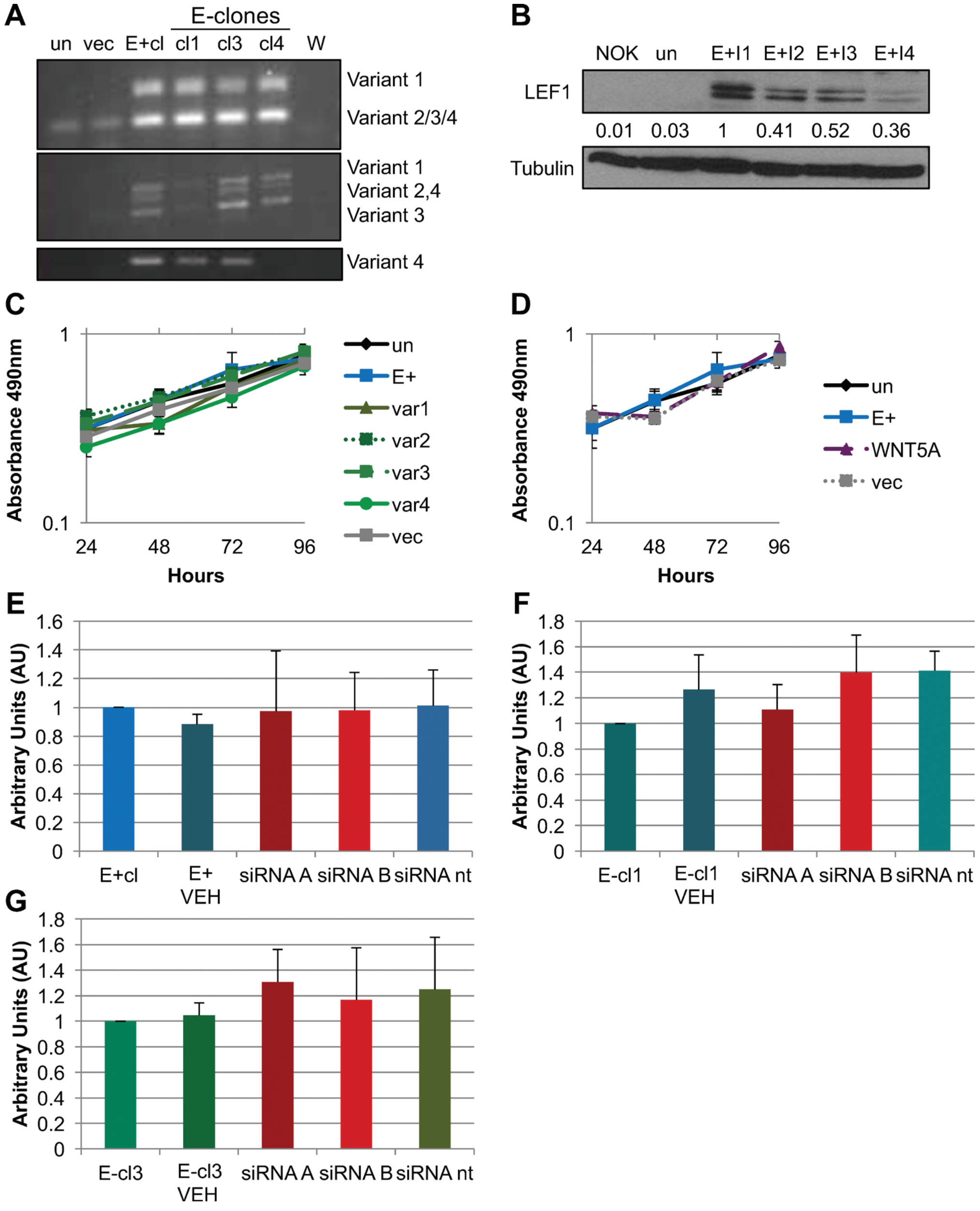

Supplementary Figure 3: Increased LEF1 mRNA variants occurs after EBV infection of NOK without affecting proliferation. (**A**) Representative images of RT-PCR for LEF1 mRNA variants 1–4. Primers were designed to distinguish isoforms by size based on exon use. PCR products were visualized on a 1% agarose gel stained with ethidium bromide. (**B**) Representative western blot for LEF1 in EBV-positive cells lines. Tubulin is shown as a loading control. Values are the average signal intensity of four biological replicates normalized to tubulin with E+I1 set to 1. Proliferation assays for stable NOK cell lines expressing (**C**) LEF1 and (**D**) WNT5A. Values are the average of at least three biological replicates analyzed in triplicate. Error bars are the SEM. MTS proliferation assay at 48 hours in supplement free media for LEF1 knockdown in (**E**) E+cl cells, (**F**) E-cl1 cells, and (**G**) E-cl3 cells. Shown is the average of two biological replicates analyzed in triplicate. Error bars are the SEM. Un: uninfected, E+I1/2/3/4: EBV-positive cell lines, E+cl: EBV-positive clone, E-cl1/3: EBV-negative transiently infected clones, VEH: E+ or E-cls treated with transfection reagent alone, siRNA A/B: LEF1 specific siRNA, siRNA nt: non-target siRNA, vec: empty vector control cells, E+: EBV-positive cells, var1/2/3/4: LEF1 stable cell line, WNT5A: WNT5A stable cell lines.

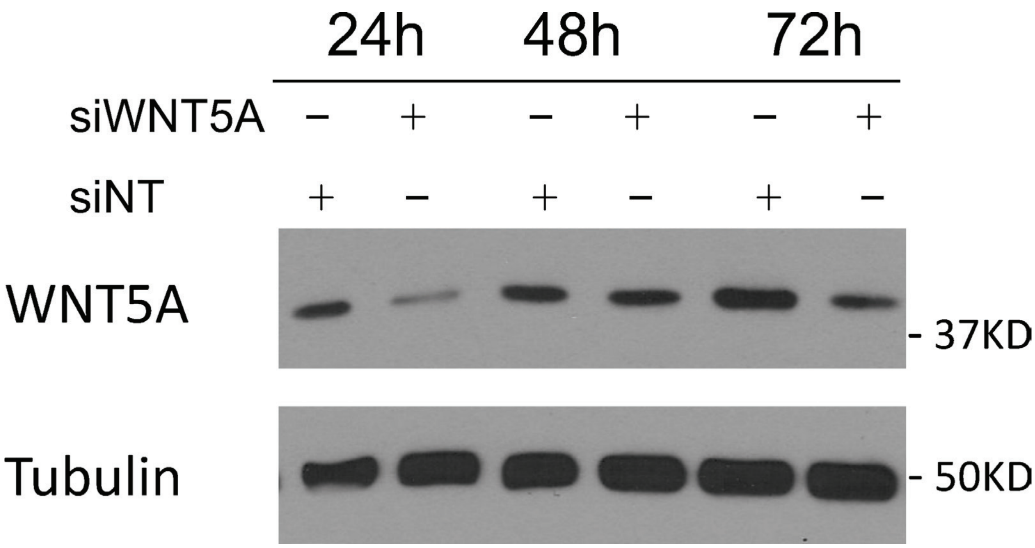

Supplementary Figure 4: Timecourse of WNT5A knockdown. A 10 nM pool of 4 WNT5A siRNAs or 10 nM nontarget siRNA was transfected into EBV-positive NOK. At 24 hour intervals, protein lysates were collected, and immunoblot analysis for WNT5A was performed.

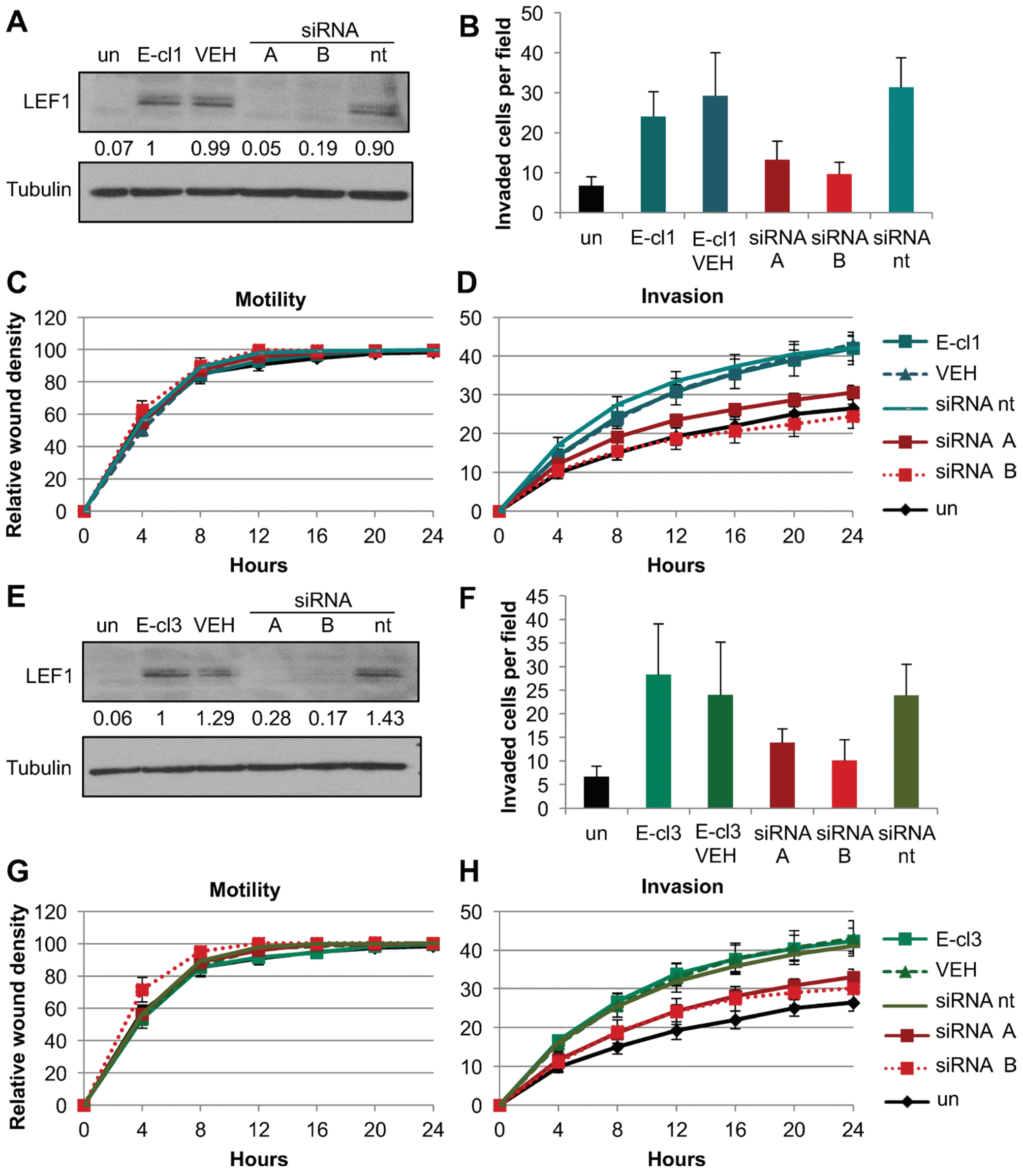

Supplementary Figure 5: EBV invasive phenotype is dependent on LEF1 in EBV-negative transiently infected clones. (**A** and **E**) Representative western blot of LEF1 knockdown in E-cl1 and E-cl3 cells, respectively. Tubulin is shown as loading control. Values are the average signal intensity of four biological replicates normalized to tubulin with E-cl1/3 set as 1. (**B** and **F**) Transwell invasion assay at 24 hours in E-cl1 and E-cl3 cells, respectively. Shown is the average of two biological replicates analyzed in duplicate. 5 images were analyzed for invaded cells per 10× field per insert. Error bars are the SEM. (**C** and **G**) Wound healing motility assay in E-cl1 and E-cl3 cells, respectively. Shown is the average of four biological replicates analyzed in duplicate and error bars are the SEM. (**D** and **H**) Wound healing invasion assay for E-cl1 and E-cl3 cells, respectively. Shown is the average of four biological replicates analyzed in duplicate and error bars are the SEM. Un: uninfected cells, VEH: E-cl1/3 treated with transfection reagent alone, siRNA A/B: LEF1 siRNA, siRNA nt: non-target siRNA, E-cl1/3: EBV-negative transiently infected clones.

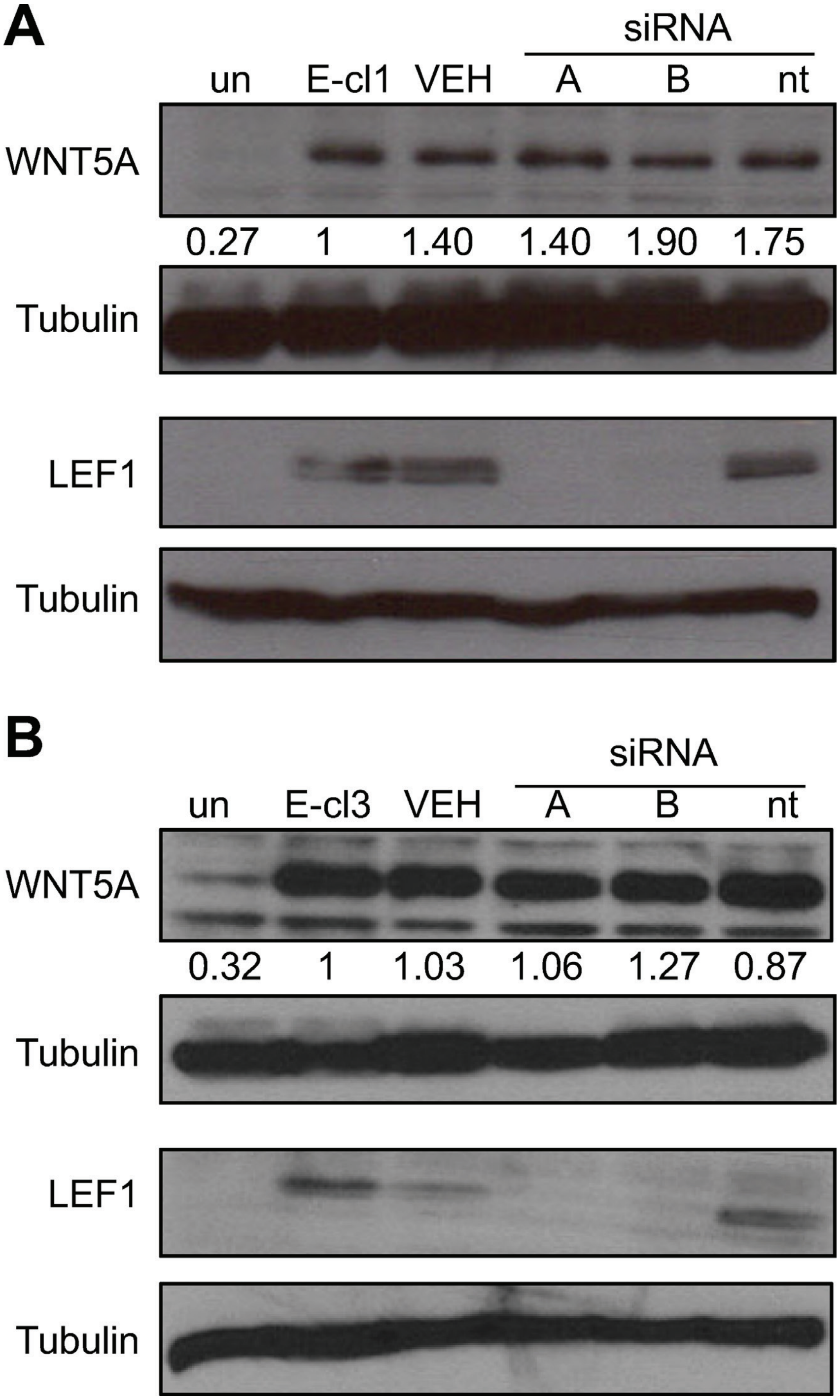

Supplementary Figure 6: LEF1 knockdown does not alter WNT5A protein levels in EBV-negative transiently infected clones. Representative western blots for WNT5A and LEF1 following LEF1 knockdown in (**A**) E-cl1 cells and (**B**) E-cl3 cells. Tubulin is shown as a loading control. Numbers are the average signal intensity of three biological replicates normalized to tubulin with E-cl1 and E-cl3 set to 1. Un: uninfected, E-cl1/3: EBV-negative transiently infected clone 1/3, VEH: E-cls treated with transfection reagent alone, siRNA A/B: LEF1 specific siRNA, siRNA nt: non-target siRNA.

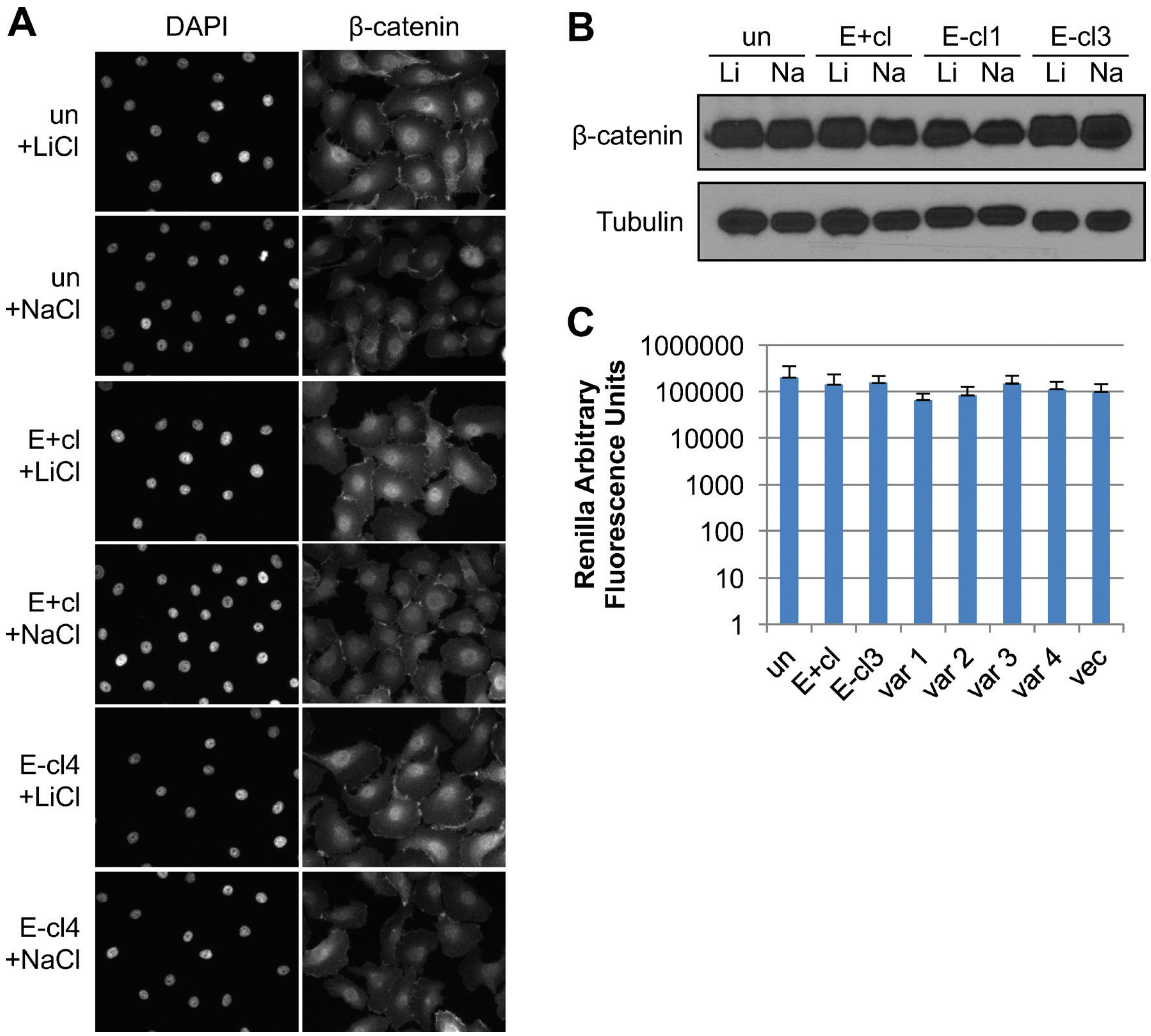

Supplementary Figure 7: EBV infection does not affect β-catenin levels and nuclear accumulation after LiCl treatment. (**A**) Representative immunofluorescence for β-catenin (right panels) and DAPI (left panels) in cells treated with 20 mM lithium chloride (LiCl) or sodium chloride (NaCl) for 6 hours. (**B**) Representative western blot for β-catenin in LiCl or NaCl treated cells. Tubulin is shown as a loading control. (**C**) The average values for renilla fluorescence used to normalize the LEF1 reporter assay is shown. Un: uninfected cells, E+cl: EBV-positive clone, E-cl4: EBV-negative transiently infected clone 4, LiCl: lithium chloride, NaCl: sodium chloride.

Supplementary Table 1: Primers used in this study

gene	Ref	F	R	Position	Accession#
LEF1		CTCCTGCTCCTT TCTCTGTTC	ATCCCTCATCCA GCTATTGTAAC	1961-1983/2068-2048	NM_016269.4
WNT5A		TGCCAGTATCAA TTCCGACATC	GCTCACCGCGTA TGTGAAG	967-988/1083-1065	NM_003392.4
Involucrin		TCCTCCAGTCAATACCCATCAG	CAGCAGTCATGTGCTTTTCCT	118-139/225-245	NM_005547.2
Cyclophilin A		GCAGGAACCCTTATAACCAAATCC	CTTGGGCCGCGTCTCC	239-216/131-146	NM_021130.3
hHPRT		CGTCTTGCTCGAGATGTGATG	TTTATAGCCCCCCTTGAGCAG	309-329/386-367	NM_000194.2
BRLF1	Wille CK, *et al* J Virol. 2013	AATCTCCACACTCCCGGCTGTAAA	TGGCTTGGAAGACTTTCTGAGGCT	9276492787/92878-92855	AJ507799
BZLF1	Wille CK, *et al* J Virol. 2013	TTGGGCACATCTGCTTCAACAGGA	AATGCCGGGCCAAGTTTAAGCAAC	89990-90013/90181-90158	AJ507799
EBNA1	Ryan JL, *et al* J Mol Diagn. 2004	CCGCTCCTACCTGCAATATCA	CAATAACGGCAGCAAGCTTG	97421-97441/98221-98202	AJ507799
LMP2A	Ryan JL, *et al* J Mol Diagn. 2004	CGACCCCATATCGCAACACT	CGTGCCATTGCTGTGGAAG	34-57/579-556	AJ507799

Supplementary Table 2: Antibodies and plasmids

Antibodies

Protein	Cat #	Company	Dilution
LEF1	C12A5	Cell Signaling	Western 1:1000, IF 1:100
WNT5A	MAB645	R&D	Western 1:500, IF 1:100
WNT5A/B	C27E8	Cell Signaling	Western 1:1000
Tubulin	sc-8035	Santa Cruz	Western 1:10,000
β-catenin	sc-7199	Santa Cruz	Western 1:2000, IF 1:200
Involucrin	sc-28557	Santa Cruz	Western 1:1000
Actin	sc-1616	Santa Cruz	Western 1:10,000
P-AKT	9271	Cell Signaling	Western 1:1000
AKT	2920	Cell Signaling	Western 1:1000
P-ERK	4370	Cell Signaling	Western 1:1000
ERK	4696	Cell Signaling	Western 1:1000
IκBα	4812	Cell Signaling	Western 1:1000
p65	sc-109	Santa Cruz	Western 1:500
p65	sc-8008	Santa Cruz	IF 1:50
Tubulin (for fluorescent western blot)	sc-32293	Santa Cruz	Western 1:10,000

Plasmids

Name	Cat #	Company	Reference
LEF1 var1	RC208663	Origene	
LEF1 var2	RC225565	Origene	
LEF1 var3	RC225588	Origene	
LEF1 var4	RC228237	Origene	
pCMV6	PS100001	Origene	
WNT5A	35911	Addgene	Najdi *et al* Differentiation. 2012
pcDNA		Invitrogen	
Super8xTOPFLASH	12456	Addgene	Veeman *et al.* Curr Biol. 2003
Super8xFOPFLASH	12457	Addgene	Veeman *et al.* Curr Biol. 2003
